# Supplementary material for: Influence of Light Spectra from LEDs and Scion × Rootstock Genotype Combinations on the Quality of Grafted Watermelon Seedlings
Source: Plants (Basel). 2021 Feb 12;10(2):353. doi: 10.3390/plants10020353 (PMC7918498; doi:10.3390/plants10020353)
Supplement: Supplementary file 1 [file plants-10-00353-s001.pdf]

Influence of light spectra from LEDs and scion × rootstock genotype combinations on the quality of grafted watermelon seedlings - Supplementary

Supplementary Table S1. Analysis of variance for the effect of light treatment and watermelon × interspecific squash (W×S) genotype combination on important qualitative characteristics of grafted watermelon seedlings. R/S: root-to-shoot dry weight ratio; Chl content: relative chlorophyll content; DW/L: shoot dry weight-to-length ratio; DQI: Dickson's quality index; a*: red/green coordinate; b*: yellow/blue coordinate.

Parameter	Light	W×S	Light * W×S
Shoot length	***	ns	***
Stem diameter	***	ns	**
Leaf area	***	ns	ns
Shoot dry weight	*	**	ns
Root dry weight	ns	**	**
R/S ratio	ns	ns	*
DW/L	**	*	**
DQI	*	*	***
Chl content	**	ns	**
Lightness	***	ns	**
Chroma	***	ns	*
Hue angle	***	ns	ns
a*/b*	***	ns	ns

ns: non-significant

*, **, and ***: significant difference at $p \leq 0.050$, $p \leq 0.010$, and $p \leq 0.001$, respectively

Supplementary Table S2. Colorimetric parameters of grafted watermelon seedlings derived from four watermelon × interspecific squash genotype combinations, and after illumination by seven light treatments during healing. a*: red/green coordinate; b*: yellow/blue coordinate. Mean values (± SE) within a scion × rootstock genotype combination followed by different letters are significantly different ($p \leq 0.05$).

Light treatment	Lightness	Chroma	Hue angle	a*/b*
**Celine F1 × TZ-148**				
R	41.28 ± 0.53 a	25.65 ± 0.85 a	128.10 ± 0.48 a	-0.78 ± 0.01 a
B	41.06 ± 0.39 a	26.01 ± 0.95 a	128.53 ± 0.42 a	-0.80 ± 0.01 a
36B	40.29 ± 0.85 a	25.84 ± 1.05 a	129.15 ± 0.43 a	-0.81 ± 0.01 a
24B	40.37 ± 0.34 a	24.50 ± 0.66 a	129.60 ± 0.47 a	-0.83 ± 0.01 a
12B	40.47 ± 0.75 a	24.29 ± 0.87 a	129.62 ± 0.40 a	-0.83 ± 0.01 a
12B+FR	40.95 ± 0.63 a	26.21 ± 1.50 a	128.89 ± 0.57 a	-0.81 ± 0.02 a
W	41.55 ± 0.45 a	25.36 ± 0.85 a	128.31 ± 0.30 a	-0.79 ± 0.01 a
**Sunny Florida F1 × TZ-148**				
R	40.35 ± 0.63 b	26.79 ± 0.86 ab	128.42 ± 0.41 ab	-0.79 ± 0.01 ab
B	42.63 ± 0.74 a	29.69 ± 1.54 a	127.32 ± 0.54 b	-0.76 ± 0.02 a
36B	39.57 ± 0.43 b	25.10 ± 0.43 b	129.15 ± 0.25 a	-0.81 ± 0.01 b
24B	39.20 ± 0.55 b	24.65 ± 0.76 b	129.32 ± 0.46 a	-0.82 ± 0.01 b
12B	39.18 ± 0.35 b	24.93 ± 0.51 b	129.09 ± 0.21 a	-0.81 ± 0.01 b
12B+FR	41.09 ± 0.46 ab	26.78 ± 0.57 ab	128.50 ± 0.36 ab	-0.80 ± 0.01 ab
W	41.14 ± 0.32 ab	27.06 ± 0.47 ab	128.30 ± 0.33 ab	-0.79 ± 0.01 ab
**Celine F1 × Radik**				
R	41.28 ± 0.24 ab	26.69 ± 0.59 b	128.50 ± 0.27 bc	-0.80 ± 0.01 b
B	43.10 ± 0.48 a	30.67 ± 1.04 a	127.23 ± 0.34 c	-0.76 ± 0.01 a
36B	40.87 ± 0.62 b	26.62 ± 1.05 b	128.87 ± 0.38 ab	-0.81 ± 0.01 bc
24B	39.22 ± 0.50 b	23.05 ± 0.46 c	129.84 ± 0.16 a	-0.83 ± 0.01 c
12B	39.67 ± 0.33 b	26.22 ± 0.60 bc	128.67 ± 0.34 ab	-0.80 ± 0.01 bc
12B+FR	39.28 ± 0.65 b	26.03 ± 0.74 bc	128.72 ± 0.29 ab	-0.80 ± 0.01 bc
W	39.31 ± 0.34 b	24.75 ± 0.53 bc	129.21 ± 0.23 ab	-0.82 ± 0.01 bc
**Sunny Florida F1 × Radik**				

R	40.81 ± 0.77 a	24.98 ± 1.06 b	128.88 ± 0.31 a	-0.81 ± 0.01 b
B	41.86 ± 0.68 a	29.78 ± 0.80 a	127.01 ± 0.25 b	-0.75 ± 0.01 a
36B	41.14 ± 0.79 a	26.56 ± 1.57 ab	128.88 ± 0.72 a	-0.81 ± 0.02 b
24B	41.01 ± 0.59 a	26.39 ± 0.64 ab	128.56 ± 0.24 ab	-0.80 ± 0.01 ab
12B	40.38 ± 0.32 a	26.78 ± 0.76 ab	128.80 ± 0.36 a	-0.80 ± 0.01 ab
12B+FR	40.95 ± 0.35 a	25.41 ± 0.66 b	129.18 ± 0.25 a	-0.82 ± 0.01 b
W	40.24 ± 0.48 a	25.58 ± 0.89 b	128.59 ± 0.43 ab	-0.80 ± 0.01 ab
